# Enhancing nutritional and sensory properties of plant-based beverages: a study on chickpea and Kamut® flours fermentation using *Lactococcus lactis*

**DOI:** 10.3389/fnut.2024.1269154

**Published:** 2024-01-24

**Authors:** Marina Mefleh, Ghofrane Omri, Rosangela Limongelli, Fabio Minervini, Monica Santamaria, Michele Faccia

**Affiliations:** Department of Soil, Plant, and Food Sciences, University of Bari Aldo Moro, Bari, Italy

**Keywords:** fermentation, *Lactococcus lactis*, plant-based beverage, minerals, raffinose, viscosity, consumer acceptance, alternative proteins

## Abstract

The study aimed to set up a protocol for the production of a clean-label plant-based beverage (PBB), obtained by mixing chickpeas and Kamut^®^ flours and using a commercial *Lactococcus lactis* (LL) as fermentation starter, and to characterize it, from nutritional, microbiological, textural, shelf-life, and sensory points of view. The effect of using the starter was evaluated comparing the LL-PBB with a spontaneously fermented beverage (CTRL-PBB). Both PBBs were high in proteins (3.89/100 g) and could be considered as sources of fiber (2.06/100 g). Notably, *L. lactis* fermentation enhanced the phosphorus (478 vs. 331 mg/kg) and calcium (165 vs. 117 mg/kg) concentrations while lowering the raffinose content (5.51 vs. 5.08 g/100 g) compared to spontaneous fermentation. Cell density of lactic acid bacteria increased by *ca.* two log cycle during fermentation of LL-PBB, whereas undesirable microbial groups were not detected. Furthermore, *L. lactis* significantly improved the beverage’s viscosity (0.473 vs. 0.231 Pa s), at least for 10 days, and lightness. To assess market potential, we conducted a consumer test, presenting the LL-PBB in “plain” and “sweet” (chocolate paste-added) variants. The “sweet” LL-PBB demonstrated a higher acceptability score than its “plain” counterpart, with 88 and 78% of participants expressing acceptability and a strong purchase intent, respectively. This positive consumer response positions the sweet LL-PBB as a valuable, appealing alternative to traditional flavored yogurts, highlighting its potential in the growing plant-based food market.

## 1 Introduction

The European market share of plant-based food and beverage is growing exponentially with projections indicating a Compounded Average Growth Rate (CAGR) of 9–10% over the next 5–7 years (1). This surge is attributed to the increased consumers interest regarding the environmental impact of food production, the health implications of dietary choices and the significant venture capital investment in plant-based alternatives to animal-derived products (dairy products, meat, etc.…) (2). In particular, the consumers request for plant-based beverages (PBB) continues to move upward (3) with an anticipated annual market growth rate of 12.7% from 2022 to 2030 (4).

Despite the growing interest, consumers’ concerns reside in: (i) the poor nutritional profile (especially protein content) of PBB when compared with the conventional products (milk and fermented dairy beverages) and; (ii) the generally long list of added ingredients, employed to improve texture and flavor of the end-product (5, 6). Traditional and familiar plants used as protein sources, such as cereals, legumes, pseudocereals, and nuts, are still preferred by consumers over novel protein sources (e.g., fungi, insects, and algae) (2). The cultivation of old wheat varieties and legumes is gaining traction for their environmental sustainability and adaptability to marginal areas (7). Notably, old durum wheats predominantly grown in the Mediterranean region are recognized for their higher protein content compared to many other wheat varieties (8–10). Additionally, among the traditional plant sources, legumes confer the highest protein content to PBB, but they are deficient in certain sulfur amino acids (e.g., methionine and cysteine) which can be supplemented by cereal proteins (11).

However, legumes and other plant sources contain antinutritional factors (ANF) and off-flavors. Some ANFs, such as phytic acids and raffinose, are thermostable but could be reduced significantly through biotechnological processes, including fermentation. This process has been showed to lower the ANF content, enhancing protein digestibility, mineral bioavailability, and overall sensory properties of the plant-based food and beverages (12–14). The main microorganisms used in fermentation are usually from the *Lactobacillales* order, commonly referred to as lactic acid bacteria (15). Specifically, *Lactococcus lactis* is one of the main microorganisms used in fermented dairy products and is available as both commercial starter and a component of natural starters (16, 17).

*Lactococcus lactis* strains have been isolated from a variety of environmental sources (e.g., raw milk and many vegetables and fruits) (18, 19). However, it is believed that the industrial strains used in dairy productions originally inhabited plants and have been subsequently adapted to milk over time (16, 20). Plant-derived lactococci have showed efficacy in improving texture, flavor, and other sensory properties, thanks to their ability to produce many desirable compounds, in both dairy and plant-based products (21, 22).

In alignment with the United Nations’ Sustainable Development Goal 2 (“Zero hunger”) for 2030, the present study aimed to set up a protocol of production and characterize a sustainable and clean-label PBB. This beverage, created by blending chickpea and the old wheat Kamut® flours and fermented using a commercial *L. lactis* starter, was evaluated from nutritional, microbiological, textural, shelf-life, and sensory perspectives.

## 2 Materials and methods

### 2.1 Materials

Chickpeas (*Cicer arietinum*) flour was provided by Molini Bongiovanni S.P.A (Cambiano, Torino, Italy), whereas Kamut® (*Triticum turgidum* subsp. *turanicum*, cultivar Khorasan) flour was provided by Fior di Loto (Bentivoglio, Bologna, Italy). *Lactococcus lactis* ssp. *lactis* (Lyofast VMO 01) was purchased from the company Sacco S.r.l (Cadorago, Italy). Organic dark chocolate powder (Alce Nero) and table sugar was purchased from a local market. Culture media and supplements for microbiological analyses were purchased from Oxoid (Dublin, Ireland).

### 2.2 Protocol of preparation of the fermented PBB

Tap drinkable water was slowly added (ratio 4:1) to the chickpeas flour while pre-heating and slowly mixing. The slurry (1) was heated for 30 min at 70°C (73°C being the temperature of gelatinization as observed in preliminary rheological analysis) while stirring continuously. Likewise, water was slowly added (ratio 4:1) to the Kamut® flour. The slurry (2) was heated for 30 min at 60°C (62°C being the temperature of gelatinization as observed in preliminary rheological analysis) while stirring continuously. The temperatures for heating (70 and 60°C, respectively) had been preliminarily chosen based on the desired consistency of the slurries, namely a creamy texture similar to that of a conventional plain yogurt. The two slurries (1 and 2) were then mixed (in the ratio 1:1), stirred and homogenized through pulsed ultrasounds treatment (Sonoplus HD 3200, Bandelin, Berlin, Germany) (23). In detail, aliquots of 80 mL of mixed slurries were placed in containers of 100 mL and treated with the probe (KE 76, Type 3200 UW 24 kHz) with nominal power ultrasound of 100 W, a constant amplitude of 100%, maximum temperature set at 25°C, and with pulsed sonication (pulse duration of on-time: 10 s; pulse duration off-time: 5 s), for a total treatment time of 10 min. The probe, with a tip of 2 mm in diameter, was immersed in the slurry (direct sonication). During treatment, the slurry was held in ice bath to prevent any rise in temperature.

Afterward, the homogenized slurry was divided into two aliquots: a control (CTRL), not inoculated, and a thesis (LL-PBB) that was inoculated (0.05%, w/vol, corresponding to *ca.* 7 log CFU/mL) with *L. lactis* (21). Both these were incubated at 30°C for 16 h and then stored at 4°C. Analyses were carried out at the beginning (T0) and end (T1) of fermentation. In addition, LL-PBB was analyzed after 10 (T10) and 40 (T40) days of storage at 4°C.

### 2.3 Chemical analyses

Moisture content was determined using a thermal balance (MAC 110/NP, Radwag, Radom, Poland) and water activity (a_w_) was determined with Aqua Lab 4TE (Meter Group Inc., Pullman, WA, United States). The lipid content was determined by the Soxhlet method (24). Saturated fat content was determined in agreement with EEC 2568/91 (25). The ash content was determined following AACC method 08-0.1.01 (26). Protein content (N × 6.00) was determined by the Kjeldhal method (27). Essential amino acids content was determined after acid hydrolysis with 6 N HCl for 24 h at 110°C, as described by White et al. (28), and using the Water Pico-tag system. Calories from proteins were calculated using the Atwater conversion factors and expressed in kilocalories (29). For determination of mono- and di-saccharides (fructose, galactose, glucose, lactose, maltose, and sucrose), samples were analyzed by high-performance liquid chromatography coupled with refractive index detector on an Agilent apparatus (Agilent, Santa Clara, CA, United States) equipped with a Spherisorb Amino (NH2) column 80 Å, 5 mm, 4.6 mm × 250 mm (Waters, Milford, MA, United States). The separation was done under isocratic conditions as reported by Trani et al. (30), using acetonitrile–water (70, 30, v/v) as mobile phase at a constant flow rate (1.8 mL min^−1^). In addition, total carbohydrates were determined by difference, based on the results of moisture, lipid, protein, and ash, as follows:


$$\text{Total carbohydrates}\;\left(\%\right)=100–\left(\text{moisture}+\text{lipid}+\text{protein}+\text{ash}\right)$$

Total dietary fiber was determined following the AACC method 32-05.01 (31). Minerals contents (phosphorus, sodium, iron, nickel, calcium, magnesium, manganese, potassium, and selenium) were analyzed as described by Mefleh et al. (6).

pH was evaluated by means of a pH meter (Edge HI2002, Hanna Instruments, Columbus, OH, United States). Total titratable acidity (TTA) was measured according to the AACC 02-31.01 method (32). Briefly, 1 mL of PBB was mixed with 9 mL of distilled water and 3–5 drops of phenolphthalein 0.1% (w/v). The titration was carried out with a solution of NaOH (0.1 N) and TTA% was calculated by converting the volume (V) of NaOH (0.1 N) using the following equation:


$$\text{TTA}\;\left(\%\right)=\left(V_{\text{NaOH}}\times 0.1\;N\times 0.009\times 10\right)\times 100$$

wherein V_NaOH_ was the volume of NaOH in mL used for titration, 0.009 was the correction factor of NaOH, and 10 was the dilution factor.

Phytic acids and raffinose were determined using the kit K-PHYT 05/07 and Raffinose/D-Galactose Assay, respectively, following the manufacturer’s (Megazyme Ltd., Bray, Ireland) instructions.

### 2.4 Culture-dependent analysis of microbiota

Microbiological analyses were carried out on 1 mL of PBB, after serial dilutions in peptone water solution (8.5 g/L NaCl and 1 g/L peptone). Dilutions were plated using the following agar media and incubation conditions: Plate Count (PCA) (30°C, 48 h, for total mesophilic aerobic microorganisms), de Man Rogosa Sharpe (MRS) agar with cycloheximide (0.01% w/v) (30°C, 48 h, for mesophilic rod-shaped lactic acid bacteria, LAB), M17 agar supplemented with glucose solution (10% w/v) and cycloheximide (0.01%) (30°C, 48 h, for mesophilic coccus-shaped LAB), Violet Red Bile Glucose (VRBGA) (37°C, 24 h, for *Enterobacteriaceae*), Baird Parker supplemented with egg yolk-tellurite emulsion (37°C, 48 h, for staphylococci), Slanetz and Bartley (37°C, 48 h, for enterococci), and Sabouraud Dextrose agar (SDA) (30°C, 48 h, for molds and yeasts). For the enumeration of LAB, *Enterobacteriaceae*, molds and yeasts, pour plate technique was performed; instead, spread plate technique was carried out for the enumeration of staphylococci and enterococci. Cell densities were averaged and expressed as Log Colony Forming Units (CFU)/g of beverage.

### 2.5 Culture-independent analysis of bacterial biota

Total DNA was extracted from PBBs after spontaneous (CTRL) or driven (LL) fermentation, as well as from LL-PBB after 10 and 40 days of storage. Before extraction, 10 g of PBBs were homogenized with 90 mL of 0.1 mol/L sodium phosphate buffer (pH 7), through a lab blender mixer (Bag Mixer 400 P, Interscience International, France) for 3 min and centrifuged at 200 × *g*, for 5 min, at 4°C. Supernatant was collected and centrifuged at 20,000 × *g*, for 15 min, at 4°C; then supernatants were discarded and pellets used for DNA extraction.

DNA was extracted starting from 500 mg of pellet, using the FastDNA Spin Kit for Soil (MP Biomedicals, Illkrich, France), according to the manufacturer’s instructions (33).

Next generation sequencing experiments, including quality control and primary bioinformatics analysis, were performed by Genomix4life S.R.L. (Baronissi, Salerno, Italy). DNA quality control was carried out by using NanoDrop ND-1000 spectrophotometer (Thermo Scientific, Waltham, MA, United States) and Qubit Fluorometer 1.0 (Invitrogen Co., Carlsbad, CA, United States). DNA amplification was performed with the following primers: 5′-AGA GTT TGA TYM TGG CTC AG-3′ (Forward) and 5′-ATT ACC GCG GCT GCT GG −3′ (Reverse) (34), which target the hypervariable V1 and V3 region of the 16S rRNA gene. Each PCR reaction was assembled according to Metagenomic Sequencing Library Preparation (Illumina, San Diego, CA, United States). A negative control is included in the workflow, consisting of all reagents used during sample processing (16S amplification and library preparation) but not containing the sample, to exclude contamination. Libraries were quantified using Qubit fluorometer (Invitrogen Co., Carlsbad, CA, United States) and pooled to an equimolar amount of each index-tagged sample to a final concentration of 4 nM, including the Phix Control Library. Pooled samples were subjected to cluster generation and sequenced on MiSeq platform (Illumina, San Diego, CA, United States) in a 2 × 300 paired-end format. The generated raw sequence files (fast files) underwent quality control analysis by means of FastQC tool.

Unidentified, contaminant (plastidic and mitochondrial) and singletons OTUs were eliminated. The bioinformatic taxonomic classification of 16S rRNA targeted amplicon reads was carried out through a high-performance implementation of the Ribosomal Database Project (RDP) Classifier, described in Wang et al. (35). The Chao1 indices were calculated using the open-source metagenomics RAST server (36).

The 16S rRNA gene sequences are available in the Sequence Read Archive of NCBI (accession number PRJNA1010704).

### 2.6 Rheological analysis and color measurements

Plant-based beverages were gently stirred five times prior to rheological analyses that were carried out on PBBs maintained at 4°C. Flow curves were obtained using Mars iQ Air HAAKE, molecular advance rheometer system fitted with a Couette measuring geometry with a diameter of 25 mm. The shear rate varied from 0.00185 to 116 s^−1^ (37) and shear stress was registered at increasing shear rate. Continuous shear was applied with a delay time of 5 s between measurements at a given shear rate. Strain oscillation frequency sweep was also performed. Frequencies ranged from 0.01 to 9 Hz and the applied strain was 0.00412, which fell within the linear viscoelastic region previously determined by running a strain sweep test (37). The elastic and viscous modulus (G′ and G″) and loss tangent (tan δ) were registered as function of frequency. All results were processed using HAAKE RheoWin data manager, an internal software.

Instrumental color evaluations of lightness (L*), green-red (a*), and blue-yellow (b*) were performed using a CM-600d Konica Minolta (Chiyoda, Tokyo, Japan) colorimeter.

### 2.7 Consumer test

Forty-seven people, aged between 18 and 64 years, were randomly selected and asked to participate in the test, performed at the E. Quagliarello Campus at the University of Bari, Italy. Participants were asked to fill in a questionnaire and to try two products: (1) plain LL-PBB, with no added ingredients and (2) sweet LL-PBB, with added chocolate paste. The latter was prepared as follow: 17% of sugar and 8% of dark chocolate powder were added to the LL-PBB, and mixed until well homogenized. The two products were prepared at the plant of Vallefiorita (Ostuni, BR, Brindisi). Participants were given information about the study aims and the products to try. All participants acknowledged an informed consent statement in order to participate in the test. 22 participants were BSc students, 12 were PhD students, and 13 were researchers/professors. Before tasting, participants had to respond to four questions (Supplementary Figure 1) in order to understand better their dietary choices. Samples of the two products were served chilled at 5°C in small plastic cups. Participants assessed acidity, viscosity, color, and aroma of the products, based on nine-point hedonic face scale (38) where 1 is “terrible,” 2 is “very bad,” 3 is “bad,” 4 is “just a little bad,” 5 is “maybe good or maybe bad,” 6 is “just a little good,” 7 is “good,” 8 is “very good,” and 9 is “great.” Subsequently, participants were asked to express, on a 1–9 range scale, their overall acceptability and interest to buy the product. They were asked to start with the plain LL-PBB and then to taste the sweet LL-PBB.

No human ethics committee or formal documentation process is available. We confirm that appropriate protocols for protecting the rights and privacy of all participants were utilized during the consumer test, a verbal consent of participants was obtained, and participants could withdraw from the study at any time.

### 2.8 Statistical analysis

All the data were collected from two replicate analyses carried out on three different batches of PBBs. Data were subjected to one-way ANOVA, followed by Tukey’s HSD test at α = 0.05, using the R software (39). Data from culture-dependent microbiological analyses were subjected to the Student’s T-test at *p* = 0.05.

## 3 Results

### 3.1 Nutritional composition of plant-based beverages

Fermentation of PBB with *L. lactis* (LL-PBB) led to lower (*p* < 0.05) pH, moisture, and a_w_, and to higher (*p* < 0.05) TTA than the spontaneously fermented PBB (CTRL) (Supplementary Table 1). At day 10, pH and a_w_ of LL-PBB did not significantly vary (*p* > 0.05), whereas TTA decreased (*p* < 0.05) and moisture increased (*p* < 0.05). At day 40, pH, TTA, and moisture did not significantly vary (*p* > 0.05), whereas a_w_ decreased (*p* < 0.05).

Maltose was the highest sugar among mono- and di-saccharides found in both PBBs (CTRL and LL-PBB). Fructose, galactose, and glucose were found at significantly (*p* < 0.05) lower concentrations in the LL-PBB than in the CTRL-PBB (Table 1). No differences (*p* > 0.05) were found for lipids, saturated fat, proteins, fiber, total carbohydrates and all the minerals, except for phosphorous and calcium, which were found at higher (*p* < 0.05) concentrations in the LL-PBB than in the CTRL-PBB. In addition, LL-PBB showed higher (*p* < 0.05) concentration of ash than CTRL-PBB. Both PBB had 20% of their caloric values from proteins (Table 1).

**Table 1:** Concentrations of mono-saccharides, di-saccharides, lipids, saturated fat, ash, total carbohydrates, total digestible fiber, proteins, calculated calories from proteins, and concentrations of minerals in CTRL-PBB (spontaneous) and LL-PBB after 16 h of fermentation (T1).

	T1-CTRL	T1-LL
Fructose (g/100 g)	0.056 ± 0.00a	0.010 ± 0.00b
Galactose (g/100 g)	0.055 ± 0.01a	0.033 ± 0.00b
Glucose (g/100 g)	0.100 ± 0.002a	0.040 ± 0.002b
Maltose (g/100 g)	4.410 ± 0.00a	4.560 ± 0.10a
Sucrose (g/100 g)	0.031 ± 0.00a	0.033 ± 0.00a
Lipids (g/100 g)	0.20 ± 0.00a	0.20 ± 0.0a1
Saturated fat (g/100 g)	0.10 ± 0.00a	0.10 ± 0.00a
Ash (g/100 g)	0.52 ± 0.00b	0.83 ± 0.00a
Total carbohydrates (g/100 g)	15.0 ± 0.00a	15.3 ± 0.07a
Total digestible fiber (g/100 g)	1.99 ± 0.20a	2.12 ± 0.30a
Proteins (g/100 g)	3.84 ± 0.01a	3.93 ± 0.02a
Calories from proteins (Kcal)	20 ± 0.00a	20 ± 0.00a
Phosphorus (mg/kg)	331 ± 0.35b	478 ± 0.35a
Calcium (mg/kg)	117 ± 3.54b	165 ± 4.24a
Iron (mg/kg)	9.20 ± 0.17a	9.35 ± 0.12a
Nickel (mg/kg)	0.31 ± 0.00a	0.30 ± 0.00a
Magnesium (mg/kg)	203 ± 10.2a	208 ± 7.30a
Manganese (mg/kg)	2.65 ± 0.54a	2.70 ± 0.74a
Potassium (mg/kg)	1,661 ± 40.3a	1,682 ± 19.6a

Values (mean ± standard deviation) in the same row flanked by different letters (a-b) are significantly different (*p* < 0.05) based on Tukey’s test.

The essential amino acids (histidine, isoleucine, leucine, lysine, methionine plus cystine, phenylalanine plus tyrosine, threonine, and valine) were quantified in the LL-PBB and to have a score higher than 1, except, for the limiting amino acids lysine (0.64) and methionine plus cysteine (0.32) (Supplementary Table 2).

Before fermentation, concentrations of phytic acid and raffinose in the PBBs were 0.19 and 5.72 g/100 g, respectively. At the end of fermentation, both PBBs contained lower (*p* < 0.05) concentrations of both antinutritional compounds. In detail, concentration of phytic acid in the CTRL-PBB was 0.14/100 g, not significantly different (*p* > 0.05) from the value found in the LL-PBB (0.13/100 g). On the other hand, LL-PBB contained a lower concentration (*p* < 0.05) of raffinose (5.03/100 g) than the CTRL-PBB (5.51/100 g).

### 3.2 Culturable microbiota of PBBs

Before fermentation (T0), culturable microbiota of the CTRL-PBB was largely dominated by presumptive LAB, whereas presumptive staphylococci were found at low cell density (Table 2). However, *ca.* 90% of the colonies grown on the MRS and glucose-M17 plates inoculated with CTRL-PBB diluted at 10^−5^ were catalase-positive (data not shown), suggesting that (strictly or facultatively) aerobic bacteria grew on media that are elective for LAB. After spontaneous fermentation (T1), all the microbial groups, included undesirable *Enterobacteriaceae* and staphylococci, increased (*p* < 0.05). Yeasts were not found at detectable levels both before and after fermentation. Therefore, based on the cell densities of undesired bacteria, the spontaneously fermented beverage (CTRL-PBB) was not stored for 40 days at 4°C, unlike LL-PBB.

**Table 2:** Cell densities (log CFU/g) of different microbial groups in the formulated beverage before (T0) and after (T1) 16 h of spontaneous fermentation.

Microbial group	T0	T1
Total mesophilic aerobic microorganisms	6.0 ± 0.0b	9.0 ± 0.1a
Mesophillic rod-shaped lactic acid bacteria	6.2 ± 0.2a	7.6 ± 0.1a
Mesophilic coccus-shaped lactic acid bacteria	6.1 ± 0.0b	8.0 ± 0.0a
Enterococci	<1	3.0 ± 0.0a
*Enterobacteriaceae*	<1	7.1 ± 0.1a
Presumptive staphylococci	1.3 ± 0.0b	4.8 ± 0.0a
Presumptive yeasts	<1	<1

Values were the average (± standard deviation) of three biological replicates analyzed in duplicate. Mean values in the same row flanked by different letters (a-b) are significantly different (*p* < 0.05) based on Student’s T-test.

Before fermentation of PBB with *L. lactis* (LL-PBB, T0), the culturable microbiota was dominated by LAB, as expected, whereas presumptive staphylococci were found at much lower cell density (Table 3). After fermentation (T1), cell densities of LAB and mesophilic aerobic microorganisms increased (*p* < 0.05) by *ca.* two log cycles, whereas all the other microbial groups, including staphylococci, were not detectable. After 10 days of storage at 4°C (LL-PBB, T10), cell densities of presumptive coccus-shaped LAB and mesophilic aerobic microorganisms did not vary (*p* > 0.05), whereas the number of presumptive rod-shaped LAB decreased (*p* < 0.05). No enterococci, enterobacteria, staphylococci, and yeasts were found (Table 3). After 40 days of storage (LL-PBB, T40), rod-shaped LAB further decreased (*p* < 0.05) to 5.7 log CFU/g; coccus-shaped LAB and mesophilic aerobic microorganisms also decreased (*p* < 0.05), reaching an order of magnitude of 5 log CFU/g. At this time-point of shelf-life, enterococci and enterobacteria were detected as subdominant bacterial populations, whereas staphylococci and yeasts were still not detectable (Table 3).

**Table 3:** Cell densities (log CFU/g) of different microbial groups in the formulated beverage before (T0), after 16 h of driven fermentation (T1), and during 10 (T10) and 40 (T40) days of storage at 4°C.

Microbial group	T0	T1	T10	T40
Total mesophilic aerobic microorganisms	7.3 ± 0.0b	9.1 ± 0.1a	9.0 ± 0.1a	5.5 ± 0.1c
Presumptive mesophillic rod-shaped lactic acid bacteria	7.3 ± 0.0c	9.1 ± 0.0a	8.4 ± 0.1b	5.2 ± 0.5d
Presumptive mesophilic coccus-shaped lactic acid bacteria	7.3 ± 0.0b	9.0 ± 0.1a	8.9 ± 0.0a	5.7 ± 0.1c
Presumptive enterococci	<1	<1	<1	2.9 ± 0.0a
Presumptive *Enterobacteriaceae*	<1	<1	<1	2.0 ± 0.0a
Presumptive staphylococci	1.9 ± 0.0a	<1	<1	<1
Presumptive yeasts	<1	<1	<1	<1

Values were the average (± standard deviation) of three biological replicates analyzed in duplicate. Mean values in the same row followed by different letters (a-d) are significantly different (*p* < 0.05) based on Student’s T-test.

### 3.3 Bacterial biota of PBBs described through culture-independent analysis

The metagenetic analysis of the two fermented PBBs (i.e., CTRL and LL) through 16S allowed to observe a higher species richness (number of identified species and values of Chao1 and Shannon index) in CTRL-PBB (spontaneously fermented), compared to LL-PBB (fermented by *L. lactis*) (Supplementary Table 3). CTRL-PBB microbiota was dominated by *Bacillus* sp. and *Alphaproteobacteria* (Class), which were also found, at lower percentage of relative abundance in LL-PBB (Supplementary Table 4). LL-PBB microbiota was largely dominated by *L. lactis* which, however, was detected as sub-dominant OTU also in CTRL-PBB. Moreover, the number of identified species and values of Chao1index tended to increase during shelf-life of LL-PBB (Supplementary Table 3). However, during 40 days of shelf-life, the relative abundances of bacterial OTUs found in the LL-PBB did not vary with respect to day 1 (Supplementary Figure 2).

### 3.4 Texture and color measurements

Plant-based beverage fermented with *L. lactis* was characterized by significantly (*p* < 0.05) higher values for all the viscosity parameters (except for the loss tangent, ratio G″/G′, which was not significantly different), compared to CTRL-PBB (Table 4). During storage of LL-PBB, viscosity decreased by 30% after 10 days and by 45% after 40 days. Both G′ and G″ decreased (*p* < 0.05) after 10 days and then, after 40 days, they did not vary (*p* > 0.05).

**Table 4:** Rheological measurements and color parameters of CTRL-PBB (spontaneous) and LL-PBB after 16 h of fermentation (T1), and after 10 (T10-LL) and 40 (T40-LL) days of storage at 4°C.

	T1-CTRL	T1-LL	T10-LL	T40-LL
Viscosity (Pa∙s)	0.231 ± 0.00d	0.473 ± 0.00a	0.344 ± 0.00b	0.260 ± 0.00c
Shear (Pa)	26.6 ± 0.36d	54.5 ± 0.48a	39.7 ± 1.10b	30.0 ± 0.33c
G′ (Pa)	109 ± 10b	265 ± 68a	85.8 ± 2.92b	113 ± 5.78b
G″ (Pa)	31.5 ± 4.20b	80.6 ± 7.76a	22.0 ± 0.91b	26.9 ± 0.48b
G″/G′	0.29 ± 0.07a	0.31 ± 0.05a	0.26 ± 0.02a	0.23 ± 0.02a
L^*^	58.6 ± 0.04c	74.3 ± 0.05a	74.5 ± 0.08a	70.7 ± 0.01b
a^*^	0.65 ± 0.28a	0.39 ± 0.17b	0.31 ± 0.06b	0.28 ± 0.03b
b^*^	16.1 ± 0.32b	19.2 ± 0.39a	19.3 ± 0.11a	16.7 ± 0.11b

Mean values in the same row flanked by different letters (a-d) are significantly different (*p* < 0.05) based on Tukey’s test. L*: lightness, a*: red/green, b*: yellow/blue.

Regarding the color indexes, LL-PBB showed significantly (*p* < 0.05) higher values of L* (lightness) and b* (yellow), and lower value of a* (red) compared to CTRL-PBB (Table 4). The differences in terms of color indexes between the *L. lactis* fermented and the spontaneously fermented PBBs could be clearly perceived in the Supplementary Figure 3. After 10 days of storage of LL-PBB, no signifcant (*p* > 0.05) differences were found in the values of color indexes, with respect to the end of fermentation, whereas after 40 days L* and b* significantly (*p* < 0.05) decreased.

### 3.5 Consumer test

Participants who claimed to follow a vegan and flexitarian diets were 2 and 6%, respectively. 53% of participants reported to rarely buy vegan food and 32% reported to never buy them. 90% of participants expressed their interest in buying healthy food (data not shown).

More than 50% of participants rated positively (score ≥ 6) the viscosity and color of the plain LL-PBB, but only 38% of participants gave positive scores to acidity. When participants tasted the sweet (added with chocolate paste) LL-PBB, all the attributes were rated positively by at least 62% of participants. In detail, the percentages almost doubled for acidity and flavor, with respect to those observed for the plain LL-PBB (Supplementary Table 5).

Acceptability of the plain LL-PBB was rated positively by 46% of participants (Supplementary Figure 4A); the percentage increased to 88% for the sweet LL-PBB (Supplementary Figure 4C). 58% of the participants expressed good interest in buying the plain LL-PBB (Supplementary Figure 4B), and this percentage increased to 78% for the sweet LL-PBB (Supplementary Figure 4D).

## 4 Discussion

The use of the commercial starter *L. lactis* ssp. *lactis* VMO 01 in this study markedly influenced various parameters, notably acidification, water activity, nutrients concentrations, and raffinose levels, compared to the spontaneously fermented beverage. The pH levels observed in the LL-PBB align with those reported in other legumes-based fermented beverages (21, 40). Distinctively, LL-PBB demonstrated a lower pH and higher total titratable acidity (TTA) compared to the control (CTRL-PBB). This was evidenced by the reduced residual concentrations of monosaccharides, likely consumed more extensively via lactic acid fermentation by the lactococcal starter. The resultant higher acidity was found to be pleasantly balanced when chocolate paste was added, enhancing the overall taste profile. The lower moisture found in the LL-PBB is consistent with a study on soymilk fermentation (41) and could be explained by the increase in dry matter content observed as lactic acid fermentation proceeds.

Contrasting with the results reported by Mefleh et al. (21), the LL-PBB demonstrated higher ash concentrations compared to the CTRL-PBB, paralleling the observed increases in phosphorus and calcium levels. While an initial hypothesis was that the higher concentration of those two minerals in LL-PBB could be attributed to reduced levels of phytic acid, a known antinutritional factor that limits mineral bioavailability (42), our results did not indicate significant differences in the reduced phytic acid between the CTRL and the *L. lactis* fermented beverages. This phenomenon might be explained by the activation of endogenous phytases in the flour at the reduced pH levels, as cereal phytases show the highest activity at pH 5.5, but remain active even at lower pH values (43). In order to explain the higher concentration of minerals found in the PBB fermented by *L. lactis*, we may propose two hypotheses: (i) the fermentation by *L. lactis* might have released higher amount of lactic acid than spontaneous fermentation, thus improving mineral solubility (44); (ii) minerals might be bound to ANF other than phytic acid, such as tannins and raffinose (42). Compared to the CTRL, a lower concentration of raffinose, a potential cause of gastrointestinal disturbances if consumed in a high quantity (> 15 g/day) (42), was noted in the LL-PBB. This could be potentially due to the raffinose-degrading capability (through α-galactosidase activity) of *L. lactis* strains (45), especially in those isolated from vegetables, such as the commercial strain used in this study. Therefore, we may hypothesize that the commercial starter used could have decreased raffinose concentration. In addition to these findings, the LL-PBB displayed several interesting nutritional traits: fat free (<0.5/100 g), source of fiber (>1.5 g of fiber/100 Kcal) (46), and a protein content similar to that of a conventional yogurt (minimum of 2.7/100 g) (47). The formulation, which includes wheat and chickpeas, achieved a complete Essential Amino Acid (EAA) profile, meeting 100% of the amino acid requirements as per guidelines of FAO/WHO/UNU (48), with the exception of methionine + cysteine and lysine. Given that casein, commonly found in dairy products, is also deficient in cysteine and methionine, the *L. lactis*-fermented PBB could be considered as a valuable alternative to fermented dairy beverages.

As expected, fermentation of PBB differently drove the microbial community depending on the use of the *L. lactis* commercial starter. In the LL-PBB, LAB were the only microbial population detected, whereas in the CTRL, LAB and other bacterial groups were found, including undesirable *Enterobacteriaceae* and staphylococci at critical values (*ca.* 5 log cfu/g or higher) of cell density. It is likely that the pH reached in the LL-PBB, lower than 4.5, inhibited the growth of undesired microorganisms. The role of LAB as tools to guarantee safety of food is well known, when used as either starters or protective cultures. In detail, *L. lactis* is used in bio-preservation because some groups of this species synthesize the bacteriocin nisin. In addition, the antibacterial activity of this species could be due to its ability to release organic acids, hydrogen peroxide, and diacetyl (49). The values of cell density of LAB in the LL-PBB fell in the range found in previous studies on legume-based beverages fermented with LAB (40, 50, 51). In agreement with the results from culture-dependent analyses, the use of the *L. lactis* commercial starter simplified the bacterial community compared to the CTRL, as shown by the lower values of all the alpha diversity indexes. As shown by 16S metagenetics analysis, the CTRL was dominated by undesirable bacterial groups. Among those groups, the spore-forming *Bacillus* sp., which includes species able to cause food-borne diseases or food spoilage (52, 53), represented the most abundant of all the bacterial OTUs detected in the CTRL-PBB. Probably, the heat treatment (60–70°C for 30 min) carried out on the flours as the first step of the protocol of preparation and the following direct sonication did not inactivate bacterial spores which, in absence of the commercial starter, could have the possibility to germinate and the resulting vegetative cells to multiply (54). On the other hand, the very high relative abundance of an OTU classified as *L. lactis* in the LL-PBB confirmed the dominance of the starter, as suggested by the results of culture-dependent analysis. It is probable that in the LL-PBB the *L. lactis* strain used as starter inhibited the growth of undesired microorganisms, presumably because of release of lactic acid and other antimicrobial compounds (55, 56).

In our findings, the *L. lactis*-fermented PBB exhibited a notably improved viscosity compared to the control (CTRL-PBB), a result that aligns with previous research on beverages fermented with LAB (21, 57). A plausible explanation for this increased viscosity is the synthesis of exopolysaccharides (EPS) by the lactococcal starter (58). Indeed, EPS are known for their natural gelling and thickening capacity (59, 60). At the end of fermentation, the measurements of the storage modulus (G′) and loss modulus (G″) of LL-PBB were comparable to those observed in a yogurt manufactured using EPS-producing strain of *S. thermophilus* (61). This similarity underscores the potential of EPS-producing lactococcal strains in enhancing the textural attributes of plant-based fermented beverages, mirroring the effects traditionally achieved in yogurts.

The LL-PBB was subjected to a consumer test to provide insights into consumer preferences and market potential for two variants of PBBs (3). The LL-PBB was presented to consumers in two variants: “plain” (without additional ingredients) and “sweet” (with added chocolate paste). The inclusion of chocolate paste was hypothesized to potentially elevate consumer appeal, especially considering the presence of chickpea flour (approximately 50% of the total flour blend) in the formulation which is known to impart a thermoresistant beany flavor not well appreciated (6). Both acidity and viscosity were appreciated in both variants, likely due to the pH reduction and the presumed synthesis of EPS by the lactococcal starter (14). Notably, the “sweet” LL-PBB variant, with added chocolate paste, showed higher acceptability and purchase interest scores than the “plain” variant. This finding suggests that the “sweet” LL-PBB could be considered as a valuable and appealing alternative to traditional flavored yogurts, particularly in segments of the market seeking novel plant-based options.

Throughout its shelf-life, notable changes were observed in the LL-PBB. Although no post-acidification was observed, LAB populations, especially mesophilic lactobacilli decreased. This observation aligns with previous studies on fermented PBBs reporting a decreased viability of LAB after 30 days of storage at 4°C (62, 63). It is postulated that a combination of storage at low temperature and low pH environment possibly induced a decline phase in the LAB populations, as supported by findings in similar studies (63, 64). Additionally, the viscosity of LL-PBB decreased over time. These results are essential for understanding the storage and distribution potential of this product. The change in viscosity could be due to partial hydrolysis of the starch-protein matrix, facilitated by both endogenous and microbial enzymes. Moreover, the acidic conditions prevailing during storage may have also contributed to the hydrolysis of the EPS matrix, further influencing the beverage’s viscosity. Such interactions between microbial activity, enzymatic processes, and storage conditions underscore the dynamic nature of the LL-PBB’s textural properties over its shelf-life.

## 5 Conclusion

This study successfully developed an environmentally friendly, clean-label PBB using flours from chickpea and the old wheat Kamut®, fermented with a commercial *L. lactis* starter. This research enhances our understanding of using *L. lactis*, a traditional dairy fermenter, in PBB production. Notably, fermentation with *L. lactis* improved the nutritional value of the beverage, increasing the phosphorus and calcium concentrations, and reducing the raffinose content. Additionally, the LL-PBB was high in fiber and had a protein content, similar to conventional yogurt, aligning with the rising consumer interest in nutritious and sustainable plant-based foods.

After fermentation with *L. lactis*, the beverage mirrored the texture qualities of milk-based fermented foods. However, during its shelf-life, the beverage experienced a decline in LAB populations and in viscosity, underscoring the need for further research to optimize storage conditions and extend the shelf-life, while maintaining desirable sensory and textural properties. We believe that conducting shelf-life analyses at only three intervals over 40 days (on days 1, 10, and 40) was limiting. More frequent assessments would have provided a more comprehensive understanding of the beverage’s stability and dynamic changes over time.

Sensory evaluation and consumer acceptability and interest in purchasing revealed that LL-PBB with added chocolate paste held market promise as an alternative to traditional flavored yogurts, especially for consumers seeking more plant-based options.

Overall, this study contributes to the growing body of knowledge on plant-based fermented foods and the consumer behavior toward innovative plant-based foods. Future research could focus on extending the shelf-life and exploring the scalability of this production protocol, which may pave the way for the introduction of this innovative PBB into the wider market. Furthermore, a larger and more diverse group of participants in a consumer test could provide more robust insights into consumer preferences and market potential.

## Data availability statement

The original contributions presented in the study are publicly available. This data can be found at: https://www.ncbi.nlm.nih.gov/sra, accession: PRJNA1010704.

## Ethics statement

Ethical approval was not required for the study involving humans in accordance with the local legislation and institutional requirements. Written informed consent to participate in this study was not required from the participants or the participants’ legal guardians/next of kin in accordance with the national legislation and the institutional requirements.

## Author contributions

MM: Methodology, Validation, Formal analysis, Investigation, Writing – original draft, Writing – review & editing. GO: Formal analysis, Investigation, Writing – review & editing. RL: Formal analysis, Investigation, Writing – review & editing. FM: Methodology, Validation, Investigation, Writing – review & editing, Visualization, Supervision. MS: Formal analysis, Writing – review & editing. MF: Methodology, Validation, Writing – review & editing, Supervision, Project administration.
