# Supplementary material for: Enhancing nutritional and sensory properties of plant-based beverages: a study on chickpea and Kamut® flours fermentation using Lactococcus lactis
Source: Front Nutr. 2024 Jan 24;11:1269154. doi: 10.3389/fnut.2024.1269154 (PMC10847596; doi:10.3389/fnut.2024.1269154)
Supplement: Supplementary file 1 [file Table_1.pdf]

Supplementary Table 1. Moisture, water activity (a_w), total titratable acidity (TTA), and pH of CTRL (spontaneous fermentation) and LL plant-based beverage (PBB) after 16 h of fermentation (T1), and after 10 (T10-LL) and 40 (T40-LL) days of storage at 4 °C. Values (mean ± standard deviation) in the same row flanked by different letters (a-c) are significantly different ($p \leq 0.05$) based on Tukey's test.

	**T1-CTRL**	**T1-LL**	**T10-LL**	**T40-LL**
Moisture (g/100 g)	78.8±0.33b	76.0±0.14c	79.8±3.76a	79.1±0.19ab
a_w	0.995±0.00a	0.990±0.00b	0.994±0.00ab	0.984±0.00c
TTA (%)	0.540±0.00c	0.925±0.035a	0.700±0.03b	0.815±0.06ab
pH	5.14±0.02a	4.42±0.02b	4.38±0.03b	4.39±0.02b

Supplementary table 2. Concentrations of essential amino acids contents in mg g^{-1} of proteins in the PBB after fermentation with *Lactococcus lactis* (T1-LL), the essential amino acids requirements in mg g^{-1} Proteins for adults (FAO/WHO/UNU, 1991) and the calculated amino acids score. Tryptophane was not analyzed.

	**T1-LL**	**Reference (mg/g protein) - older child, adolescent, adult**	**Amino Acids Score**
Histidine	37.66	16	2.35
Isoleucine	37.66	30	1.26
Leucine	75.32	61	1.23
Lysine	30.79	48	0.64
Methionine plus cystine	7.38	23	0.32
Phenylalanine plus tyrosine	97.71	41	2.38
Threonine	30.28	25	1.21
Valine	45.80	40	1.15

Supplementary table 3. Alpha diversity indexes of *Bacteria* (16S rRNA) found in plant-based beverage (PBB) after 16 h of spontaneous (T1-CTRL) and driven (T1-LL) fermentation, and in PBB-LL after ten (T10) and forty (T40) days of storage at 4 °C. a and b indicate two technical replicates.

**Sample**	**Number of reads**	**Number of identified species**	**Chao1**	**Shannon**
T1-CTRL_a	193,838	386	1167	1.011
T1-CTRL_b	193,813	412	824	1.046
T1-LL_a	132,488	26	37	0.636
T1-LL_b	131,176	34	40	0.646
T10-LL_a	149,545	30	44	0.650
T10-LL_b	152,303	43	34	0.614
T40-LL_a	148,074	63	46	0.645
T40-LL_b	155,912	43	46	0.636

Supplementary table 4. Relative abundance (%) (mean of two replicates ± standard deviation) of bacterial OTUs, with number of reads ranged from ca. 131,000 to ca. 194,000 per sample, found in plant-based beverages after 16 h of spontaneous (T1-CTRL) and driven (T1-LL) fermentations.

**Bacterial OTUs**	**T1-CTRL**	**T1-LL**
Bacteria (Kingdom)	6.18±0.44	0.12±0.03
Chthonomonas/Armatimonadetes sp.	4.28±0.08	<0.1
Bacillales (Order)	0.16±0.01	-*
Bacillus sp.	45.62±3.14	0.19±0.03
Alkalilactibacillus ikkensis	0.32±0.01	<0.1
Lactobacillus sp.	0.71±0.00	<0.1
Lactococcus sp.	0.60±0.16	3.97±0.41
Lactococcus lactis	3.28±2.36	95.22±0.54
Proteobacteria (Phylum)	0.90±0.09	-
Alphaproteobacteria (Class)	33.70±1.66	0.32±0.04
Maricaulis sp.	0.15±0.00	-
Pleomorphobacterium xiamenense	0.85±0.01	<0.1
Ralstonia solanacearum	0.16±0.02	-
Neisseria sp.	0.27±0.03	-
Other bacteria**	3.16±0.01	0.18±0.03

*OTU not found

**Sum of relative abundances of those bacterial OTUs found at less than 0.1%

Supplementary table 5. Percentage (%) of participants that rated from 6 to 9 (where 6 is “just a little good”, 7 is “good”, 8 is “very good” and 9 is “great”) the acidity, viscosity, color and flavor of the PBB fermented by *Lactococcus lactis* tasted in its plain (PBB-LL) and sweet (Sweet PBB-LL) version.

	**PBB-LL (%)**	**Sweet PBB-LL (%)**
Acidity	38	62
Viscosity	52	70
Color	86	100
Flavor	44	82
