# Supplementary material for: Enhancing nutritional and sensory properties of plant-based beverages: a study on chickpea and Kamut® flours fermentation using Lactococcus lactis
Source: Front Nutr. 2024 Jan 24;11:1269154. doi: 10.3389/fnut.2024.1269154 (PMC10847596; doi:10.3389/fnut.2024.1269154)

Supplementary Figure 1. Questionnaire given to the participants of the consumer test

Questions	Answers
What type of diet do you follow?	a- Mediterranean<br>b- Vegan<br>c- Vegetarian<br>d- Flexitarian<br>e- Other
Do you consider buying 'healthy' product when doing the grocery?	a- Always<br>b- Frequently<br>c- Rarely<br>d- Never
Do you consider buying 'vegan' product?	a- Always<br>b- Frequently<br>c- Rarely<br>d- Never
How do you rate your legumes consumption?	a- Once per month<br>b- Twice a month<br>c- Once per week<br>d- Twice or more per week

Supplementary Figure 2. Relative abundance (%) of bacterial OTUs found in plant-based beverage after 16 h of driven (T1-LL) fermentation, and after ten (T10-LL) and forty (T40-LL) days of storage at 4 °C.

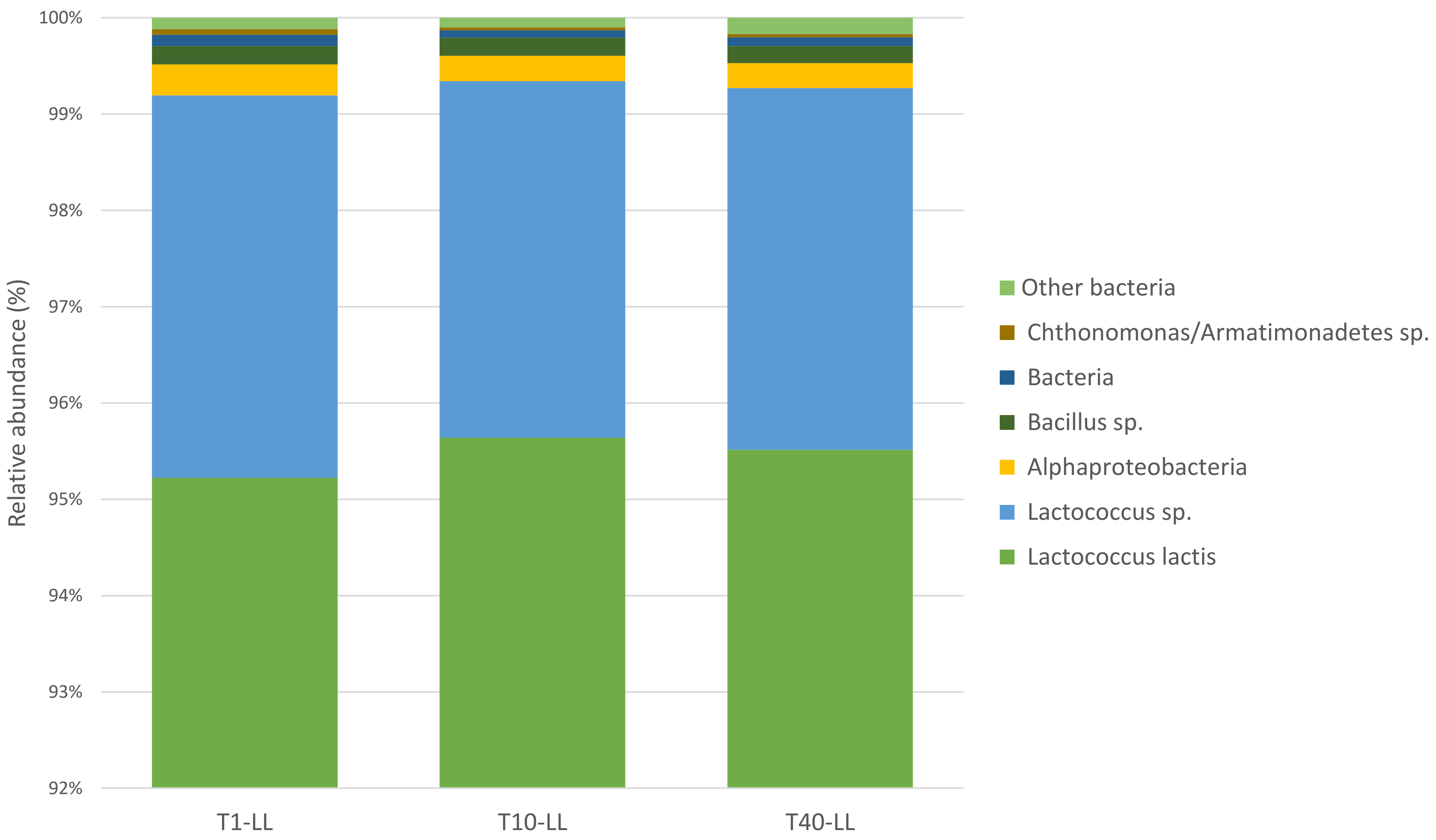

Supplementary Figure 3. Appearance of PBBs after 16 h of driven (L. lactis, A) or spontaneous (CTRL, B) fermentation.

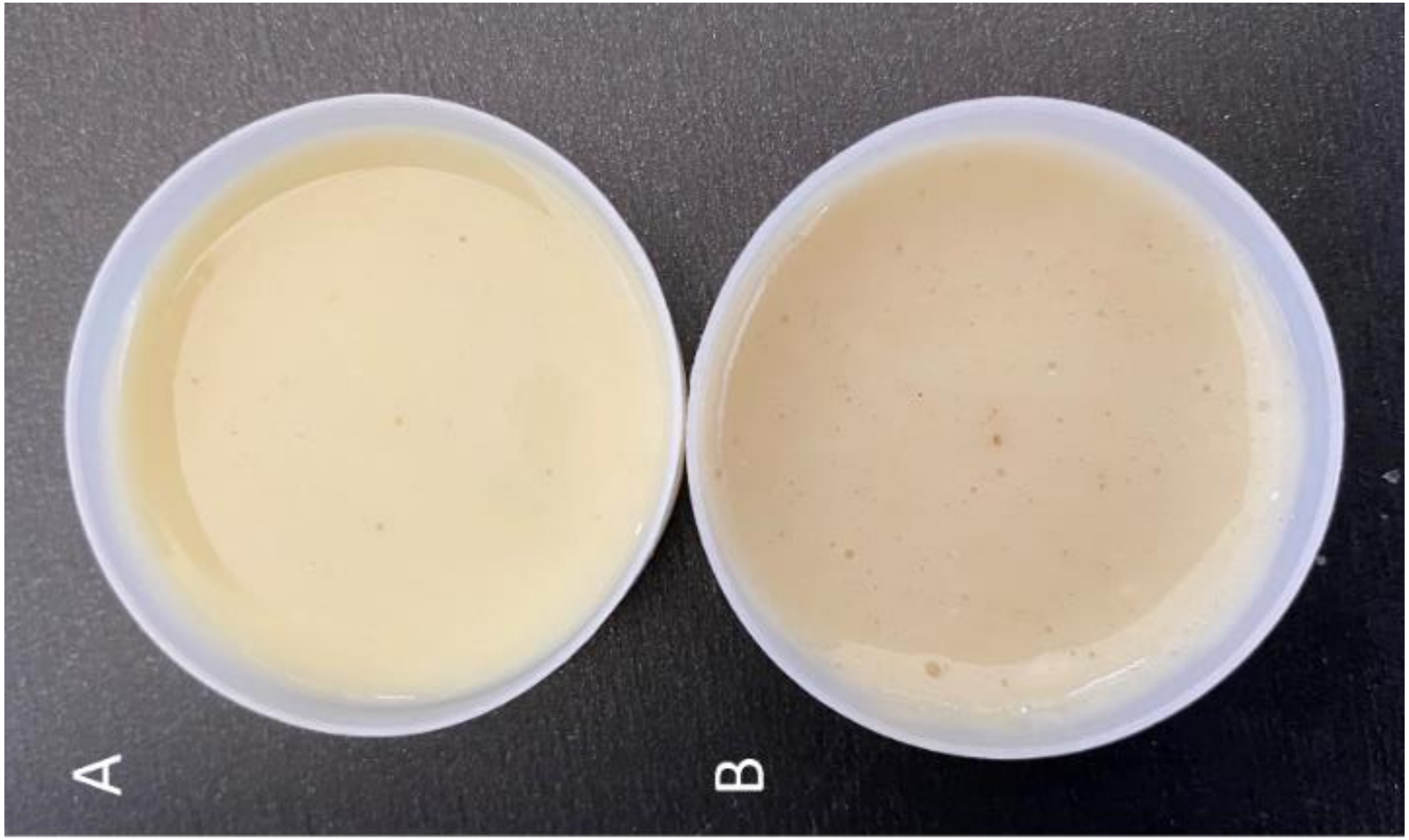

Supplementary Figure 4. Pie charts describing, on a scale from 1 to 9, acceptability for the plain PBB (panel A) and the sweet PBB (panel C), and interest in buying the plain PBB (panel C) and the sweet PBB (panel D).

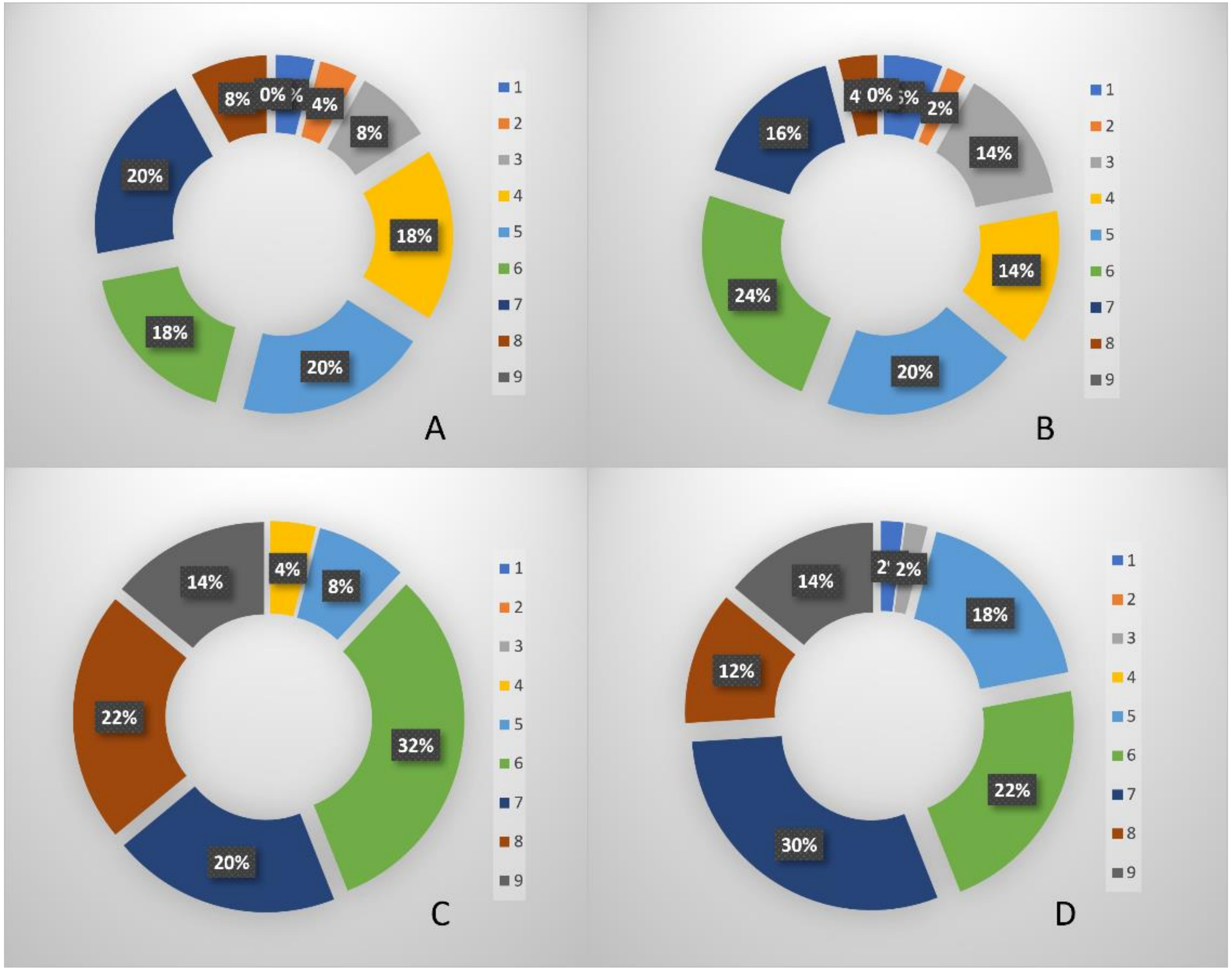

Supplement: Supplementary file 2 [file Presentation_1.pdf]
